# Association between the 21-gene recurrence score and isolated locoregional recurrence in stage I-II, hormone receptor-positive breast cancer

**DOI:** 10.1186/s13014-020-01640-1

**Published:** 2020-08-17

**Authors:** David D. Yang, Daniela L. Buscariollo, Angel M. Cronin, Shicheng Weng, Melissa E. Hughes, Richard J. Bleicher, Adam L. Cohen, Sara H. Javid, Stephen B. Edge, Beverly Moy, Joyce C. Niland, Antonio C. Wolff, Michael J. Hassett, Rinaa S. Punglia

**Affiliations:** 1 Harvard Medical School, 25 Shattuck St, Boston, MA 02115 USA; 2 Department of Radiation Oncology, Dana-Farber/Brigham and Women’s Cancer Center, 75 Francis St, Boston, MA 02115 USA; 3 Division of Population Science, Dana-Farber Cancer Institute, 450 Brookline Ave, Boston, MA 02215 USA; 4 Department of Medical Oncology, Dana-Farber/Brigham and Women’s Cancer Center, 75 Francis St, Boston, MA 02115 USA; 5 Department of Surgical Oncology, Fox Chase Cancer Center, 333 Cottman Ave, Philadelphia, PA 19111 USA; 6 Department of Medicine, Division of Oncology, Huntsman Cancer Institute, 1950 Circle of Hope Dr, Salt Lake City, UT 84112 USA; 7 Department of Surgery, University of Washington School of Medicine, Box 356410, Seattle, WA 98105 USA; 8 Department of Surgical Oncology, Roswell Park Cancer Institute, 665 Elm St, Buffalo, NY 14203 USA; 9 Department of Medical Oncology, Massachusetts General Hospital, 55 Fruit St, Boston, MA 02114 USA; 10 Department of Diabetes and Cancer Discovery Science, City of Hope Comprehensive Cancer Center, 1500 East Duarte Road, Duarte, CA 91010 USA; 11 Department of Oncology, Johns Hopkins University Sidney Kimmel Comprehensive Cancer Center, 401 N. Broadway, Weinberg, Baltimore, MD 21231 USA

**Keywords:** Breast cancer, Locoregional recurrence, Recurrence score

## Abstract

**Background:**

Although the 21-gene recurrence score (RS) assay is widely used to predict distant recurrence risk and benefit from adjuvant chemotherapy among women with hormone receptor-positive (HR+) breast cancer, the relationship between the RS and isolated locoregional recurrence (iLRR) remains poorly understood. Therefore, we examined the association between the RS and risk of iLRR for women with stage I-II, HR+ breast cancer.

**Methods:**

We identified 1758 women captured in the national prospective Breast Cancer-Collaborative Outcomes Research Database who were diagnosed with stage I-II, HR+ breast cancer from 2006 to 2012, treated with mastectomy or breast-conserving surgery, and received RS testing. Women who received neoadjuvant therapy were excluded. The association between the RS and risk of iLRR was examined using competing risks regression.

**Results:**

Overall, 19% of the cohort (*n* = 329) had a RS ≥25. At median follow-up of 29 months, only 22 iLRR events were observed. Having a RS ≥25 was not associated with a significantly higher risk of iLRR compared to a RS < 25 (hazard ratio 1.14, 95% confidence interval 0.39–3.36, *P* = 0.81). When limited to women who received adjuvant endocrine therapy without chemotherapy (*n* = 1199; 68% of the cohort), having a RS ≥25 (*n* = 74) was significantly associated with a higher risk of iLRR compared to a RS < 25 (hazard ratio 3.66, 95% confidence interval 1.07–12.5, *P* = 0.04). In this group, increasing RS was associated with greater risk of iLRR (compared to RS < 18, hazard ratio of 1.66, 3.59, and 7.06, respectively, for RS 18–24, 25–30, and ≥ 31; *P*_trend_ = 0.02).

**Conclusions:**

The RS was significantly associated with risk of iLRR in patients who did not receive adjuvant chemotherapy. The utility of the RS in identifying patients who have a low risk of iLRR should be further studied.

## Background

Gene expression profiling has emerged as a useful clinical tool for patients with early-stage breast cancer. One such gene expression assay, the 21-gene recurrence score (RS), has been demonstrated to add utility to traditional clinicopathologic factors for prognosticating distant recurrence risk and predicting response to adjuvant chemotherapy in patients with early-stage, hormone receptor-positive (HR+) breast cancer [1–3]. Yet, the utility of the RS in assessing the risk of isolated locoregional recurrence (iLRR) is poorly understood.

Improving our ability to prognosticate the risk of iLRR among women with early-stage breast cancer could allow for a more individualized approach to use of postoperative radiation therapy (RT), an area of active investigation. The CALGB 9434 trial found that RT decreased the 10-year locoregional recurrence from 10 to 2% for women age ≥ 70 years with early-stage breast cancer treated with breast-conserving surgery (BCS) and tamoxifen [4]. Similarly, the PRIME II trial found that RT reduced local recurrence from 4.1 to 1.3% at 5 years for women age ≥ 65 years with early-stage breast cancer treated with BCS and endocrine therapy [5]. Neither trial demonstrated improvement in metastasis-free or cancer-specific survival. Ongoing studies are investigating the use of gene expression profiling to prospectively select younger patients for whom RT may be avoided. Given the long natural history of HR+ breast cancer, results from these trials will not be available for many years. In the interim, we examined the association between the RS and risk of iLRR for women with stage I-II, HR+ breast cancer.

## Methods

**Fig. 1:**
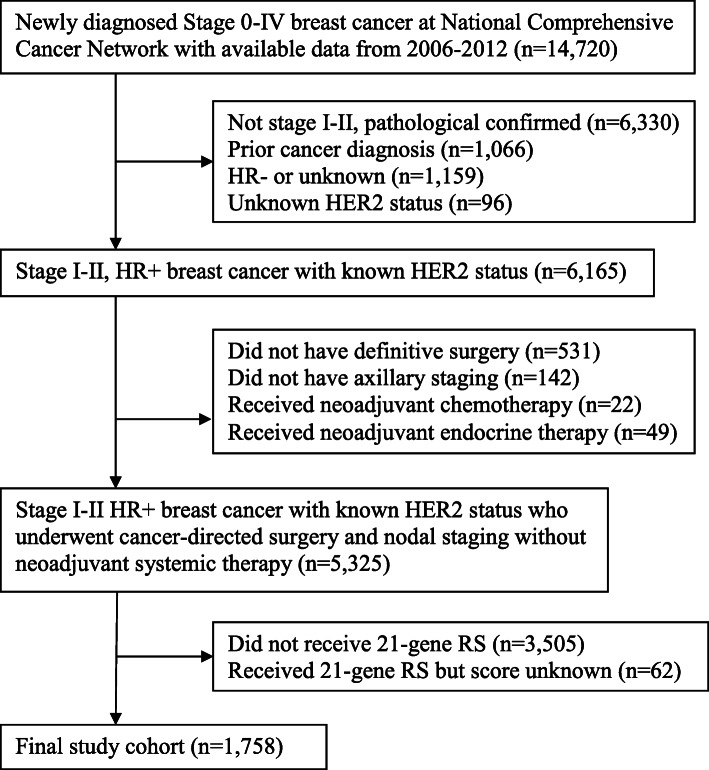
Formation of the study cohort. HR: hormone receptor. RS: recurrence score

The Breast Cancer-Collaborative Outcomes Research Database (BC-CORD) is a national, prospective database of patients with newly diagnosed breast cancer who received care at one of eight participating cancer centers in the United States. Using BC-CORD, we identified 1758 women who were diagnosed with stage I-II, HR+ breast cancer from 2006 to 2012, treated with mastectomy or BCS, and whose RS values were known (Fig. 1). Staging was determined using criteria from the American Joint Committee on Cancer (AJCC) Cancer Staging, 6th Edition [6], for patients diagnosed between 2006 through 2009, and AJCC Cancer Staging, 7th Edition, for patients diagnosed between 2010 through 2012 [7].

BC-CORD included follow-up for recurrence and survival status for all patients through January 31, 2013. Given the limited number of recurrences, additional follow-up through December 31, 2017 was obtained for 565 women treated at two institutions with records which were readily available.

Our primary analysis categorized RS as ≥25 vs. < 25. Secondary analyses categorized RS in four ordinal groups (< 18, 18–24, 25–30, ≥31). Patient characteristics were compared using Pearson’s χ^2^ test for categorical variables and the Wilcoxon rank-sum test for continuous variables. Cumulative incidence of iLRR (ipsilateral breast/chest wall or regional nodal recurrence) was estimated considering distant metastases and death as competing risks. The association between the RS and iLRR was examined using univariable competing risks regression [8]. As only 22 iLRR events were observed, the data were underpowered for multivariable analyses. To account for differences in treatment across scores, a subgroup analysis of patients treated with adjuvant endocrine therapy without chemotherapy was performed. All statistical testing was two-sided with significance set at *P* < 0.05. Statistical analyses were performed using Stata 13.1 (StataCorp, College Station, TX). Institutional review board approval was obtained before undertaking this study.

## Results

Study cohort characteristics appear in Table 1. Most patients (54%) had RS < 18; 27% had RS of 18–24, 10% had RS of 25–30, and 9% had RS ≥31. The median follow-up was 29 months (interquartile range 16–69 months). Overall, women with RS ≥25 did not have a significantly higher risk of iLRR compared to those with RS < 25 (hazard ratio [HR] 1.14, 95% confidence interval [CI] 0.39–3.36, *P* = 0.81).

**Table 1:** Baseline patient characteristics of the entire cohort

Characteristics	RS < 25	RS ≥ 25	***P***
**Total, n (%)**	1429 (81)	329 (19)	–
**Age, median (interquartile range)**	53 (47–61)	56 (48–64)	0.002
**Stage, n (%)**			0.61
I	996 (70)	234 (71)	
II	433 (30)	95 (29)	
**Surgery type, n (%)**			0.46
Breast-conserving surgery	1013 (71)	240 (73)	
Mastectomy	416 (29)	89 (27)	
**Adjuvant chemotherapy, n (%)**			< 0.001
No	1173 (82)	81 (25)	
Yes	256 (18)	248 (75)	
**Adjuvant hormonal therapy, n (%)**			0.009
No	63 (4)	26 (8)	
Yes	1366 (96)	303 (92)	
**Adjuvant chemotherapy and hormonal therapy group, n (%)**			< 0.001
Adjuvant hormonal therapy without chemotherapy	1125 (79)	74 (22)	
Both adjuvant chemotherapy and hormonal therapy	241 (17)	229 (70)	
Adjuvant chemotherapy without hormonal therapy	15 (1)	19 (6)	
Neither adjuvant chemotherapy nor hormonal therapy	48 (3)	7 (2)	
**Postoperative radiation, n (%)**			0.77
No	397 (28)	94 (29)	
Yes	1032 (72)	235 (71)	
**Number of events, n**			–
iLRR	18	4	
DM without iLRR	11	9	
Death without iLRR or DM	7	6	

*DM* distant metastasis, *iLRR* isolated locoregional recurrence, *RS* recurrence score

**Fig. 2:**
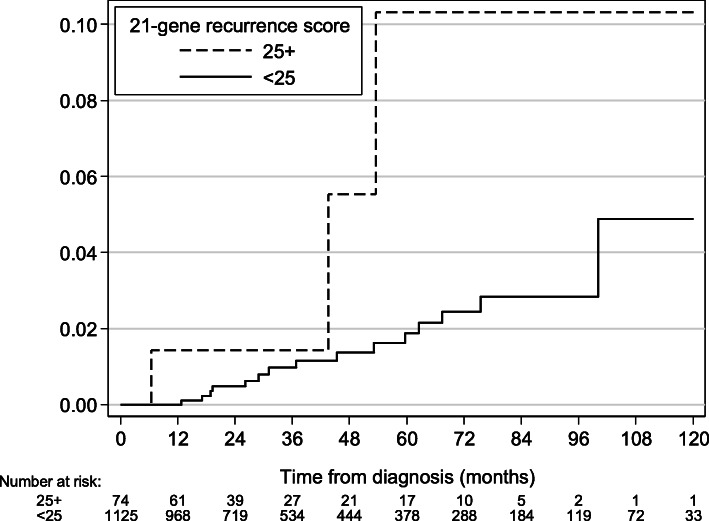
Cumulative incidence of iLRR for patients who received adjuvant endocrine therapy without chemotherapy. iLRR: isolated locoregional recurrence

Patient characteristics stratified by treatment subgroups (use of endocrine therapy and chemotherapy) are listed in Table 2. Among the 68% (*n* = 1199) of patients treated with endocrine therapy without chemotherapy, 6% (*n* = 74) had RS ≥25. In this group, with median follow-up of 30 months (interquartile range 16–72 months), RS ≥25 was associated with a significantly higher risk of iLRR (HR 3.66, 95% CI 1.07–12.5, *P* = 0.04; Table 3; 60-month cumulative incidence of iLRR 10.3% vs. 1.9%; Fig. 2). Furthermore, the risk of iLRR was significantly larger with increasing RS, with HR of 1.66, 3.59, and 7.06, respectively, for RS of 18–24, 25–30, and ≥ 31 relative to RS < 18 (*P*_trend_ = 0.02; Table 4; 60-month cumulative incidence of iLRR 1.2, 3.7, 7.3, and 33.3%, respectively).

**Table 2:** Baseline patient characteristics of patients by treatment subgroups

	Treatment subgroup								
**Adjuvant hormonal therapy**	Yes			Yes			No		
**Adjuvant chemotherapy**	No			Yes			Yes or No		
**Characteristics**	**RS < 25**	**RS ≥ 25**	***P***	**RS < 25**	**RS ≥ 25**	***P***	**RS < 25**	**RS ≥ 25**	***P***
**Total, n (%)**	1125 (94)	74 (6)	–	241 (51)	229 (49)	–	63 (71)	26 (29)	–
**Age, median (interquartile range)**	54 (48–62)	65 (54–70)	< 0.001	49 (44–56)	54 (46–62)	< 0.001	52 (46–63)	53 (45–63)	0.96
**Stage, n (%)**			0.18			0.09			0.15
I	799 (71)	58 (78)		153 (63)	162 (71)		44 (70)	14 (54)	
II	326 (29)	16 (22)		88 (37)	67 (29)		19 (30)	12 (46)	
**Surgery type, n (%)**			0.03			0.37			0.81
Breast-conserving surgery	812 (72)	62 (84)		159 (66)	160 (70)		42 (67)	18 (69)	
Mastectomy	313 (28)	12 (16)		82 (34)	69 (30)		21 (33)	8 (31)	
**Postoperative radiation, n (%)**			0.84			0.86			0.59
No	301 (27)	19 (26)		66 (27)	61 (27)		30 (52)	14 (46)	
Yes	824 (73)	55 (74)		175 (73)	168 (73)		33 (48)	12 (54)	
**Number of events, n**			–			–			–
iLRR	16	3		1	1		1	0	
DM without iLRR	9	2		2	6		0	1	
Death without iLRR or DM	7	3		0	2		0	1	

*DM* distant metastasis, *iLRR* isolated locoregional recurrence, *RS* recurrence score

**Table 3:** Competing risks regression for the association between RS and iLRR

Treatment group	Competing risk regression for iLRR		60-month cumulative incidence of iLRR (95% CI)
	HR (95% CI)	***P***	
**Entire cohort**			
RS < 25	1.0 (reference)		1.8% (1.0–3.0%)
RS ≥25	1.14 (0.39–3.36)	0.81	3.2% (1.0–7.6%)
**Hormonal therapy without chemotherapy**			
RS < 25	1.0 (reference)		1.9% (1.0–3.3%)
RS ≥25	3.66 (1.07–12.5)	0.04	10.3% (2.2–25.7%)

*CI* confidence interval, *HR* hazard ratio, *iLRR* isolated locoregional recurrence, *RS* recurrence score

**Table 4:** Competing risks regression for the association between increasing RS and iLRR for patients treated with adjuvant hormone therapy without chemotherapy

Hormonal therapy without chemotherapy	Competing risk regression for iLRR		60-month cumulative incidence of iLRR (95% CI)
	HR (95% CI)	***P***_**trend**_	
RS < 18	1.0 (reference)	0.02	1.2% (0.5–2.8%)
RS 18–24	1.66 (0.59–4.66)		3.7% (1.5–7.7%)
RS 25–30	3.59 (0.78–16.6)		7.3% (1.0–22.7%)
RS ≥31	7.05 (0.97–51.3)		33.3% (0.9–77.4%)

*CI* confidence interval, *HR* hazard ratio, *iLRR* isolated locoregional recurrence, *RS* recurrence score

For the 18 women with RS < 25 and iLRR, these iLRR occurred in the ipsilateral breast (*n* = 6), chest wall (*n* = 4), ipsilateral lymph node (*n* = 6), other locoregional lymph node (*n* = 1), or concurrently in the breast/chest wall and locoregional lymph node outside the axilla (*n* = 1). For the 4 women with RS ≥25 and iLRR, these iLRR occurred in the ipsilateral breast (*n* = 1), chest wall (*n* = 2), or ipsilateral lymph node (*n* = 1).

## Discussion

Using a prospective cohort of women with stage I-II, HR+ breast cancer, we found that a 21-gene RS ≥25 was associated with an increased risk of iLRR relative to < 25 among women who received adjuvant endocrine therapy without chemotherapy. Additionally, increasing RS was associated with higher risk of iLRR. Interestingly, a significant association between the RS and iLRR was not found for the entire cohort, likely because patients who received both adjuvant endocrine therapy and chemotherapy had a very low risk of iLRR (Table 2).

Strengths of our study include use of prospectively collected data from multiple institutions, which broadens the generalizability of the results. Our results support other reports of associations between RS and risk of locoregional recurrence [9–13]. Several limitations of our study should be noted. First, our study had a relatively short follow-up of 29 months. Given the long natural history of HR+ breast cancers with locoregional recurrences which can occur beyond 5–10 years [14], our results should be interpreted with this limitation in mind. Second, there was a small number of iLRR events, which precluded multivariable adjustment for differences in treatment characteristics. To address this limitation, we performed a subgroup analysis of women who receive adjuvant endocrine therapy without chemotherapy, as chemotherapy use is directly influenced by RS. However, there were only 3 recurrences in the subgroup of patients who were treated with endocrine therapy without chemotherapy with RS ≥25. Additionally, detailed pathologic information including the presence of lymphovascular invasion, extranodal extension, and pathologic nodal staging, as well as information on why certain patients did not receive chemotherapy or endocrine therapy, were not available.

While data from ongoing trials studying omission of RT in patients selected by gene expression profiling are ongoing, our results may offer some insights into identifying patients with HR+ breast cancer who have a very low risk of developing locoregional recurrences and therefore may not benefit in a clinically meaningful way from RT. It is important to note that given the low number of locoregional recurrences in our cohort, as well as large majority having received adjuvant RT, conclusions regarding the value of the RS in predicting benefit from adjuvant RT cannot be drawn from our cohort. Other investigators have investigated whether the RS is predictive of benefit from RT using the Surveillance, Epidemiology, and End Results (SEER) database and the National Cancer Data Base (NCDB) [15]. Dong et al. examined the association of adjuvant RT with breast cancer-specific survival using a cohort of 13,246 patients from SEER with early-stage breast cancer treated with BCS [16]. They found that receipt of RT was associated with improved breast cancer-specific survival only for patients with intermediate RS. Goodman et al. investigated the association of PMRT with overall survival for patients with pT1–2 N1 estrogen receptor-positive breast cancer using cohorts from the NCDB and SEER [17]. They found that PMRT was associated with improved overall survival only for patients with a low RS. Lastly, Zhang et al. studied the association between PMRT and both breast cancer-specific survival using a cohort from SEER for patients with pT1–2N1mic estrogen receptor-positive disease and did not have a significant association, regardless of the RS [18]. It is important to note that in these 3 studies, data on locoregional recurrences were not available, and as such, these studies do not shed light on the predictive value of RT on locoregional recurrence.

## Conclusions

The RS was significantly associated with risk of iLRR in patients with early-stage, hormone receptor-positive breast cancer who received adjuvant endocrine therapy but not chemotherapy. The utility of the RS in identifying patients who have a low risk of iLRR should be further studied.

## Data Availability

The datasets used and/or analyzed during the current study are available from the corresponding author on reasonable request.

## Abbreviations

BC-CORD: Breast Cancer-Collaborative Outcomes Research Database
BCS: Breast-conserving surgery
CI: Confidence interval
HR: Hazard ratio
HR+: Hormone receptor-positive
iLRR: Isolated locoregional recurrence
LRR: Locoregional recurrence
RS: Recurrence score
RT: Radiation therapy
